# Radiogenomic Mapping of Edema/Cellular Invasion MRI-Phenotypes in Glioblastoma Multiforme

**DOI:** 10.1371/journal.pone.0025451

**Published:** 2011-10-05

**Authors:** Pascal O. Zinn, Bhanu Majadan, Pratheesh Sathyan, Sanjay K. Singh, Sadhan Majumder, Ferenc A. Jolesz, Rivka R. Colen

**Affiliations:** 1 Department of Genetics, M.D. Anderson Cancer Center, University of Texas, Houston, Texas, United States of America; 2 Department of Clinical Neurosciences, University Hospital (CHUV BH19-110), Lausanne, Switzerland; 3 Department of Radiology, Brigham and Women's Hospital, Harvard Medical School, Boston, Massachusetts, United States of America; Institut Gustave Roussy, France

## Abstract

**Background:**

Despite recent discoveries of new molecular targets and pathways, the search for an effective therapy for Glioblastoma Multiforme (GBM) continues. A newly emerged field, radiogenomics, links gene expression profiles with MRI phenotypes. MRI-FLAIR is a noninvasive diagnostic modality and was previously found to correlate with cellular invasion in GBM. Thus, our radiogenomic screen has the potential to reveal novel molecular determinants of invasion. Here, we present the first comprehensive radiogenomic analysis using quantitative MRI volumetrics and large-scale gene- and microRNA expression profiling in GBM.

**Methods:**

Based on The Cancer Genome Atlas (TCGA), discovery and validation sets with gene, microRNA, and quantitative MR-imaging data were created. Top concordant genes and microRNAs correlated with high FLAIR volumes from both sets were further characterized by Kaplan Meier survival statistics, microRNA-gene correlation analyses, and GBM molecular subtype-specific distribution.

**Results:**

The top upregulated gene in both the discovery (4 fold) and validation (11 fold) sets was *PERIOSTIN (POSTN)*. The top downregulated microRNA in both sets was miR-219, which is predicted to bind to *POSTN*. Kaplan Meier analysis demonstrated that above median expression of *POSTN* resulted in significantly decreased survival and shorter time to disease progression (P<0.001). High *POSTN* and low miR-219 expression were significantly associated with the mesenchymal GBM subtype (P<0.0001).

**Conclusion:**

Here, we propose a novel diagnostic method to screen for molecular cancer subtypes and genomic correlates of cellular invasion. Our findings also have potential therapeutic significance since successful molecular inhibition of invasion will improve therapy and patient survival in GBM.

## Introduction

Microarray technology is a novel method that allows for the simultaneous analysis of whole genome gene- and microRNA expression events [1], [2]. This latest breakthrough technology heralds an age of large-scale discovery of new and effective therapeutic strategies for Glioblastoma Multiforme (GBM). However, despite the discovery of many new molecular targets and pathways [3], [4], the search for an effective therapy continues.

Besides the multi-resistant character of GBM [5], [6], therapy failure is thought to occur due to the cancer's invasive nature and high migratory potential preventing complete surgical resection and evading current therapeutic strategies [7], [8], [9]. Furthermore, therapy is complicated by intra- and intertumoral molecular and cellular heterogeneity that is now known to cause some tumors to respond to a specific drug while another demonstrating resistance [4], [10]. Based on this recent awareness, focus is shifting toward a more personalized treatment approach. In this regard, in particular, microRNAs are being increasingly studied. MicroRNAs are non-protein coding small RNAs that serve as negative gene regulators by binding to a specific sequence in the 3′UTR of a target gene, thus regulating gene expression. A single microRNA potentially targets hundreds of genes; thus, microRNAs were found to have important roles as tumor suppressors and oncogenes [11], as well as regulators of various cancer-specific cellular features, such as proliferation, invasion, and metastasis [12], [13], [14].

Although certain genetic analyses, such as O6-methylguanine-DNA-methyltransferase (MGMT) promoter methylation and isocitrate dehydrogenase 1 (IDH1) status [4], [15], [16], [17], are frequently used in clinical practice, large scale gene- and microRNA based cancer characterization is commonly not performed due to high cost, time and manpower required for data analysis and interpretation [18]. In order for personalized medicine to transpire, a cost-effective biomarker that accurately reflects underlying molecular cancer compositions is urgently needed. Imaging, specifically MRI, is a promising biomarker that can reflect underlying tumor pathology and biological function. It can evaluate the *entire* tumor, including its peritumoral regions which harbor microscopic invasion of cancer cells [19] and which typically cannot be surgically removed and thus are rarely analyzed in the laboratory. It follows that if imaging phenotypes of GBM obtained from routine clinical MRI studies can be associated with specific gene and microRNA expression signatures, imaging phenotypes will serve as non-invasive surrogates for cancer genomic events and provide important information as to the diagnosis, prognosis, and optimal treatment. Furthermore, it will allow for bi-directional imaging phenotype-genotype correlations and discoveries. Thus, a new field termed radiogenomics has emerged and links specific MRI radiophenotypes with gene expression profiles [20].

In the past, several studies compared gene expression and MRI findings [21], [22], [23], [24], [25]. However, we present the first comprehensive radiogenomic analysis using quantitative MRI volumetrics and large-scale gene- and microRNA expression profiling in GBM.

In this study, we specifically seek to identify and corroborate relevant genomic correlates of an edema/cellular invasion GBM radiophenotype. This is of clinical significance, since cellular invasion currently is one of the major reasons for therapy failure of modern multimodal GBM treatment [9]. The discovery of targetable genes responsible for cell spread and invasion can be expected to impact modern therapy and patient survival.

## Materials and Methods

The collection of the original material and data provided by The Cancer Genome Atlas (TCGA) project was conducted in compliance with all applicable laws, regulations and policies for the protection of human subjects, and any necessary approvals, authorizations, human subject assurances, informed consent documents, and IRB approvals were obtained [26].

### Patient population

We identified 78 treatment-naïve GBM patients from TCGA whom had both gene- and microRNA expression profiles and pretreatment MR-neuroimaging. The TCGA is a publicly available resource which has produced a multi-dimensional genomic and clinical data set in GBM and other cancers [26]. Recently, MR imaging was added to this genomic repository.

### Discovery and Validation sets

To increase the robustness and validity of the analysis, the 78 patients were randomly separated into a discovery and validation set each consisting of 39 patients. By using the FLAIR signal volume as criteria for further subgrouping the patients, each set was sub-stratified into high, medium, and low FLAIR volumes (each consisting of 13 patients) corresponding to volumes of high, medium, and low peritumoral edema/invasion, respectively. High and low groups were selected for analysis and compared for differential genomic expression profiles; this resulted in a total of 52 patients (discovery set N = 26, validation set N = 26; Table 1).

**Table 1:** Discovery (N = 26) and validation (N = 26) sets.

Discovery set					Validation set				
Patient	FLAIR signal	FLAIR	Age	Gender	Patient	FLAIR signal	FLAIR	Age	Gender
D1	1	high	54	MALE	V1	1	high	17	FEMALE
D2	0.921410705	high	58	MALE	V2	0.808427556	high	73	FEMALE
D3	0.903551776	high	65	MALE	V3	0.793857093	high	79	MALE
D4	0.792396198	high	64	MALE	V4	0.754575125	high	48	MALE
D5	0.684642321	high	63	FEMALE	V5	0.740529199	high	14	MALE
D6	0.671335668	high	76	MALE	V6	0.732311458	high	55	FEMALE
D7	0.619409705	high	62	MALE	V7	0.702995687	high	69	MALE
D8	0.600450225	high	38	MALE	V8	0.633232311	high	61	MALE
D9	0.586793397	high	51	MALE	V9	0.559511598	high	57	FEMALE
D10	0.489169585	high	43	MALE	V10	0.554481874	high	30	FEMALE
D11	0.447893947	high	72	FEMALE	V11	0.544737149	high	60	MALE
D12	0.436823412	high	47	FEMALE	V12	0.473621634	high	70	MALE
D13	0.431815908	high	55	MALE	V13	0.468842522	high	69	MALE
D14	0.272961481	low	69	MALE	V14	0.190709873	low	66	MALE
D15	0.244997499	low	54	MALE	V15	0.162839492	low	64	MALE
D16	0.228409205	low	63	FEMALE	V16	0.15912111	low	71	MALE
D17	0.20064032	low	53	FEMALE	V17	0.155373587	low	71	MALE
D18	0.197133567	low	63	FEMALE	V18	0.130528034	low	63	MALE
D19	0.184682341	low	40	MALE	V19	0.128989393	low	75	FEMALE
D20	0.181475738	low	71	MALE	V20	0.123831449	low	50	MALE
D21	0.179684842	low	69	MALE	V21	0.121465206	low	17	MALE
D22	0.175007504	low	73	MALE	V22	0.117280569	low	52	FEMALE
D23	0.097268634	low	55	FEMALE	V23	0.107413451	low	60	MALE
D24	0.088534267	low	64	MALE	V24	0.075171931	low	59	FEMALE
D25	0.085067534	low	64	MALE	V25	0.059406691	low	66	FEMALE
D26	0.027853927	low	59	FEMALE	V26	0.036647045	low	76	MALE

### Image Acquisition and Analysis

All images were downloaded from the National Cancer Institute's “The Cancer Imaging Archive (TCIA)” (http://cancerimagingarchive.net/).

#### Volume Selection

**Figure 1:**
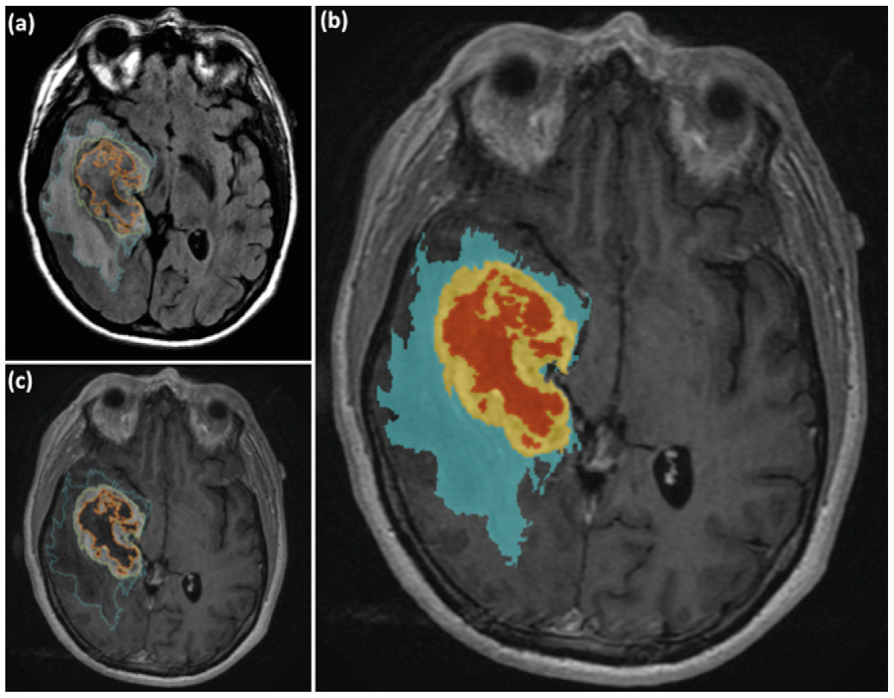
Volumetric brain tumor mapping of FLAIR, Necrosis, and Contrast Enhancement. A 55 year old male patient with a right temporal GBM. (**a**) Axial FLAIR image demonstrates segmentation (in blue) of the region of FLAIR hyperintensity corresponding to the area of edema/tumor infiltration. Notice the segmented enhancement (yellow) and necrosis (orange) that has been segmented on the T1WI post- contrast. (**b**) The segmented edema/tumor infiltration (blue), enhancement (yellow) and necrosis (orange) are seen overlaid on a base post- contrast T1WI. (**c**) Axial post-contrast enhanced T1WI demonstrates the segmentation of the enhancement (yellow) and necrosis (orange).

We used two sequences for the study: 1) Fluid-Attenuated Inversion Recovery (FLAIR) for segmentation of the edema and 2) post-contrast T1 weighted imaging (T1WI) for segmentation of enhancement (defined as tumor) and necrosis (Figure 1a, b, and c). Peritumoral FLAIR and T2 weighted image (T2WI) hyperintensity in GBM patients reflects a mixture of edema and tumor infiltration and is routinely used to evaluate its extent [19], [27], [28], [29]. Thus, these sequences were selected to compute quantitative edema/invasion volumetry. We refer to the area of peritumoral FLAIR/T2WI hyperintensity as the area of edema/invasion. In cases where FLAIR was not available for segmentation, T2WI or proton density (PD) sequences were used. In cases where post-contrast T1WI was not available, the post-contrast Spoiled Gradient Echo Recalled (SPGR) sequence was used. Standard imaging parameters were used for each of the sequences as noted in the TCGA database.

#### Image Analysis and Software

The 3D Slicer software 3.6 (http://www.slicer.org) was used for all purposes of image analysis, manipulation and segmentation. 3D Slicer is an open-source software platform developed at our institution (BWH/Harvard Medical School) for medical image processing and 3D visualization of image data [30], [31], [32]. The platform provides functionality for segmentation, registration and 3D visualization of imaging data and advanced MRI analysis algorithms. Two neuroradiologists (RRC, FAJ), with more than six (RRC) and 20 (FAJ) years of experience in image segmentation, reviewed the segmented images in consensus.

#### Image Registration, Segmentation and Model Making

To account for FLAIR and post-contrast T1WI obtained with different slice parameters, angles or voxel thickness, we rigidly aligned and registered the scans to each other. In some cases where the voxel size of the FLAIR and T1W1 series varied, we resampled the FLAIR volume to the matrix of T1W1 series. In most scans, registration was 100%. In those scans in which complex rotation modifications and registrations were required, registration was deemed adequate when error was 2 mm or less.

Segmentation was carried out in a simple hierarchical model of anatomy, proceeding from peripheral to central. Three distinct structures were segmented (edema/invasion, enhancing tumor, and necrosis). Subsequently, the models of edema, tumor and necrosis were generated from the previously performed segmentations, and the volumes of the same were automatically calculated.

### Biostatistical Image-Genomic Analysis

Affymetrix level 1 mRNA and Agilent level 2 microRNA data were downloaded from the public TCGA dataportal (April 2011) (http://cancergenome.nih.gov/). The raw data files for discovery and validation sets were separately downloaded and analyzed. Affymetrix CEL file analysis was performed in R project, a free statistical computing platform (http://www.r-project.org/) using the Bioconductor suite (http://www.bioconductor.org/). The Robust Multi-Array (RMA) algorithm was used for normalization [33].

In each patient, a total of 13,628 genes (22,267 hybridization probes) and 555 microRNAs (1,510 hybridization probes) were analyzed for significance and differential fold regulation in high versus low FLAIR groups by Comparative Marker Selection (CMS) (Broad Institute, MIT, Cambridge, MA, http://www.broadinstitute.org/cancer/software/genepattern/). CMS is a statistical method that uses permutation testing to identify differentially regulated genomic events in one versus another predefined patient group [34].

The top greater than 1.5 fold differentially expressed and concordant mRNAs and microRNAs in both the discovery and validation sets (Table S1 and S2) were then analyzed with Ingenuity Pathway Analysis (IPA) (http://www.ingenuity.com). IPA provides insight into the molecular and chemical interactions, cellular phenotypes, and disease processes of a predefined set of genes and microRNAs. By means of IPA, our top molecular targets for the high FLAIR group were analyzed in a comprehensive way and, thus, pertinent canonical pathways and functional networks accounting for edema and invasion were uncovered. Using the total amount of available TCGA patients (April 2011), the Kaplan Meier method was used to calculate overall- (N = 442) and progression-free (N = 303) survival. Mean gene expression across GBM subgroups was calculated using ANOVA and Tukey-Kramer tests. For gene and microRNA correlations we used R square statistics. All calculations were performed in Microsoft Excel 2010 and JMP 9.01 (SAS Institute, CA).

### microRNA Target Prediction

Gene target search was performed using miRWalk [35], a database providing a search function for predicted and validated microRNA targets. Both predicted and validated gene targets for the concordant microRNAs in the high FLAIR group (Table S2) were downloaded and matched with the mRNA hits from the discovery and validation sets (Table S1).

## Results

### Patient population

A total of 52 patients were included for analysis (discovery set: N = 26, 8 female, 18 male; validation set: N = 26, 9 female, 17 male) (Table 1).

### Imaging Analysis

**Figure 2:**
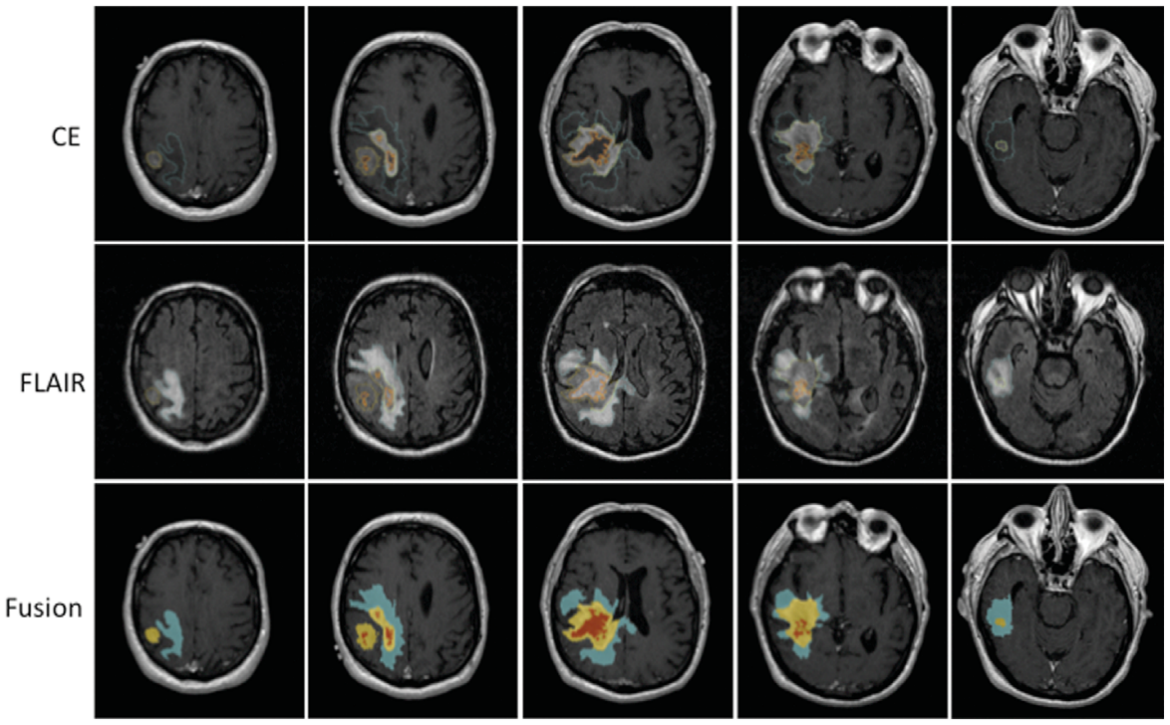
Low FLAIR patient. A 67 year old male patient with a right temporo-parieto-occipital GBM. The segmented edema/tumor infiltration (blue), enhancement (yellow) and necrosis (orange) are seen overlaid on a base post- contrast T1WI. These images demonstrate a patient with a GBM and a large amount of peritumoral FLAIR hyperintensity reflecting edema and tumor infiltration.

**Figure 3:**
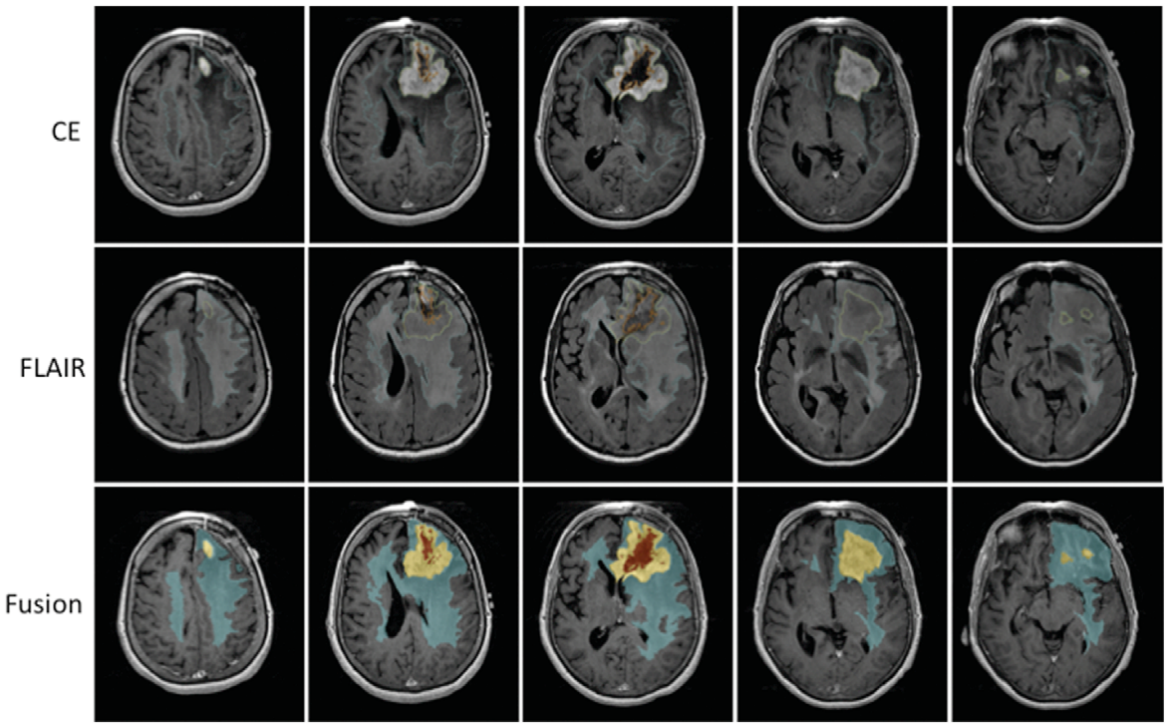
High FLAIR patient. A 59 year old female patient with a left frontal GBM. The segmented edema/tumor infiltration (blue), enhancement (yellow) and necrosis (orange) are seen overlaid on a base post- contrast T1WI. The images demonstrate a patient with a medium to large GBM with a relatively small amount of peritumoral FLAIR hyperintensity reflecting edema and tumor infiltration.

**Figure 4:**
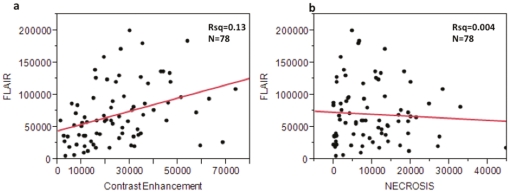
Correlation scatter plots. (**a**) FLAIR vs. Contrast Enhancement and (**b**) FLAIR vs. Necrosis volumes.

The average FLAIR volumes in the discovery and validation sets were 82,646 mm^3^ and 68,209 mm^3^ and did not significantly differ from each other (P = 0.85). The mean FLAIR volume of the discovery set's low and high FLAIR group was 33,271 mm^3^ and 132,021.5 mm^3^, respectively. The mean FLAIR volumes in the validation set's low and high groups were 20,705.5 mm^3^ and 118,651.6 mm^3^ (Figure 2 and 3, Table 1). There was an overall weak positive correlation between FLAIR and contrast enhancement volumes (R^2^ = 0.13, Figure 4a). There was no correlation between the FLAIR and necrosis volumes (R^2^ = 0.004, Figure 4b).

### Imaging-Genomic Correlation

Gene-expression analysis in high versus low FLAIR groups revealed top genes and microRNAs greater than 1.5 fold differentially and concordantly regulated in the discovery and validation sets. A total of 53 mRNAs (32 high expression; 21 low expression) and 5 microRNAs (3 high expression; 2 low expression) were identified (Table S1 and S2).

**Figure 5:**
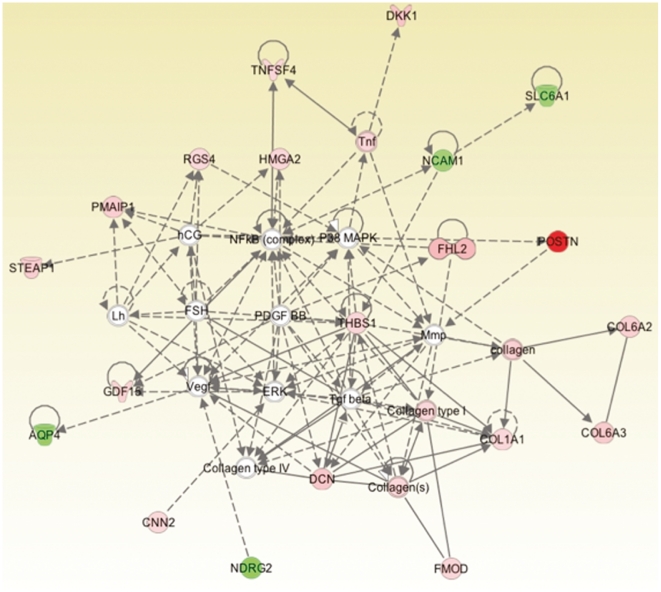
Ingenuity Pathway Analysis top network for mRNA.

**Figure 6:**
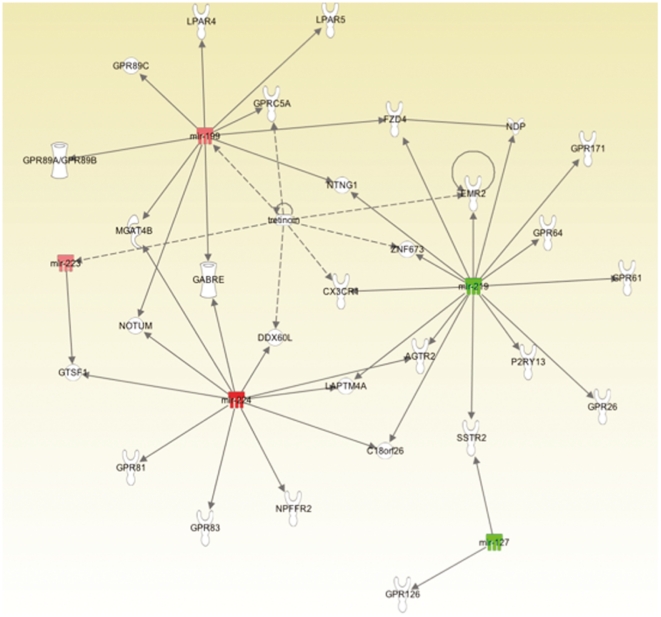
Ingenuity Pathway Analysis top network for microRNA.

**Figure 7:**
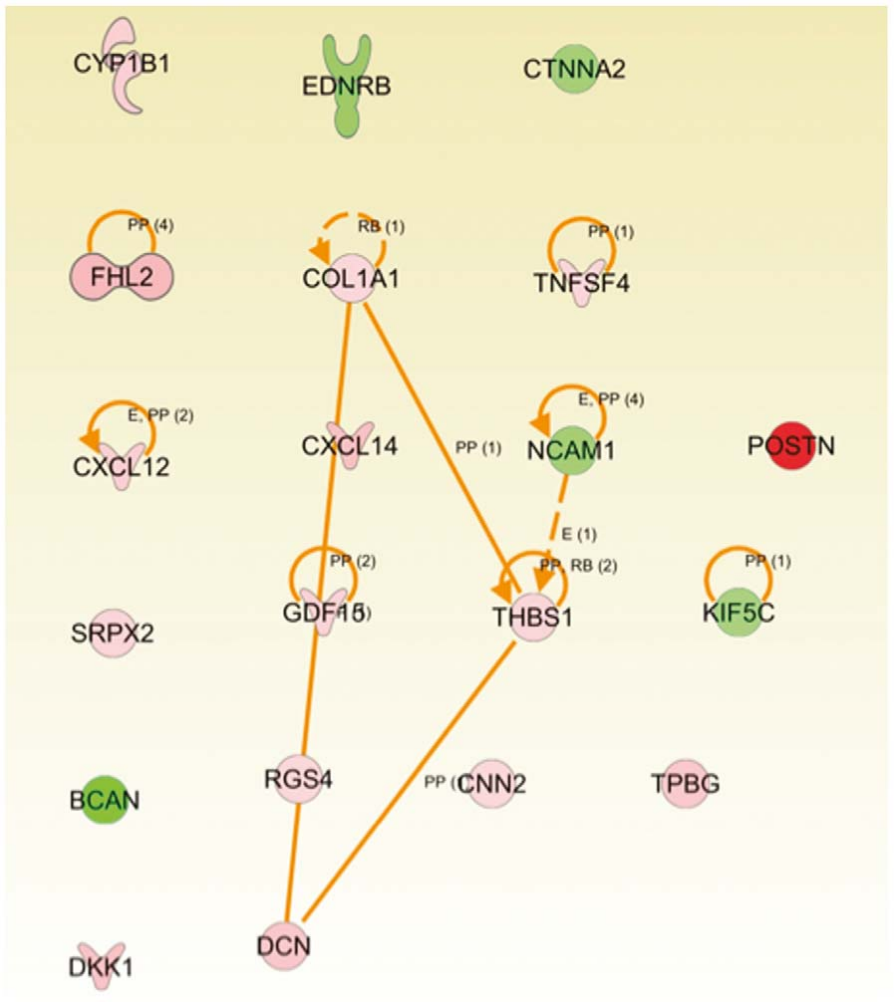
Top functional mRNA network for Cellular Movement/Invasion.

The discovered mRNA and microRNAs were separately analyzed in Ingenuity Pathway Analysis (IPA). The top mRNA disease ontology was Cancer and the top cellular functions were: Cellular Movement, Cell Morphology, and Cellular Development (Figure 5; Table 2). The top microRNA disease ontology was Cancer and the top cellular functions were: Cell Cycle, Cellular Movement, and Cell Death (Figure 6; Table 3). The top concordant mRNAs in both the discovery and validation sets involved in cellular movement and invasion (N = 20) are shown in Figure 7.

**Table 2:** Top “Diseases and Cellular functions” for mRNA.

Diseases and Disorders	P-Value	# Molecules
Cancer	2.15E-08 - 1.09E-02	29
Skeletal and Muscular Disorders	6.22E-06 - 8.22E-03	24
Genetic Disorders	7.39E-06 - 8.22E-03	38
Neurological Disease	2.57E-05 - 9.79E-03	27
Reproductive System Disease	3.36E-05 - 8.22E-03	23

Molecular and Cellular Functions	P-Value	# Molecules
Cellular Movement	1.54E-07 - 1.09E-02	20
Cell Morphology	4.41E-06 - 1.09E-02	16
Cellular Development	7.52E-06 - 1.09E-02	20
Cell-To-Cell Signaling and Interaction	2.15E-05 - 1.09E-02	13
Small Molecule Biochemistry	2.21E-05 - 1.09E-02	12

**Table 3:** Top “Diseases and Cellular functions” for microRNA.

Diseases and Disorders	P-Value	# Molecules
Cancer	3.31E-06 - 2.00E-02	3
Gastrointestinal Disease	3.31E-06 - 3.31E-06	3
Hepatic System Disease	3.31E-06 - 3.31E-06	3
Inflammatory Disease	1.15E-05 - 6.88E-03	2
Skeletal and Muscular Disorders	1.15E-05 - 1.13E-02	2

Molecular and Cellular Functions	P-Value	# Molecules
Cell Cycle	1.30E-03 - 1.30E-03	1
Cellular Movement	4.84E-03 - 4.84E-03	1
Cell Death	1.80E-02 - 1.80E-02	1

**Figure 8:**
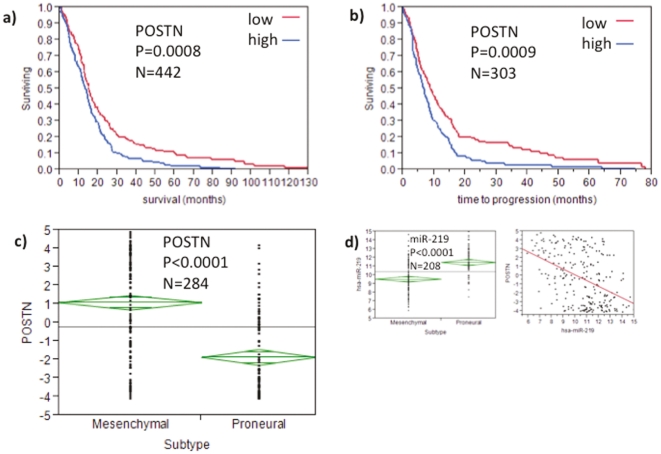
Kaplan Meier curves for Periostin. (**a**) days to death and (**b**) progression free survival. (**c**) Periostin expression levels across the two main GBM subtypes, Mesenchymal and Proneural. And (**d**) shows expression levels of miR-219 across the Mesenchymal and Proneural subtypes and in addition the inverse correlation (Rsq = 0.204) with Periostin.

The top upregulated gene in both the discovery (4 fold) and validation (11 fold) sets was *PERIOSTIN (POSTN)*. The top downregulated microRNA in both sets was miR-219, a microRNA predicted to bind to *POSTN* [35] (Figure 8a, b, and c; Table 4, S1, S2). We then examined *POSTN* expression and patient survival in the entire TCGA GBM patient cohort. Kaplan Meier analysis demonstrated that above median expression of *POSTN* resulted in significantly decreased survival (P = 0.0008, N = 442) and shorter time to disease progression (P = 0.0009, N = 303) (Figure 8a and 8b). Furthermore, we analyzed *POSTN* expression in the two most extreme GBM molecular subtypes: Mesenchymal and Proneural [36]. We found that *POSTN* is significantly upregulated in Mesenchymal GBM (P<0.0001, Figure 8c). Despite this strong association with the Mesenchymal subtype, *POSTN* expression was a stronger prognostic variable than either molecular subtype in the Cox proportional hazards ratio (*POSTN*: P = 0.03, GBM Subtype: P = 0.1; data not shown).

**Table 4:** Bioinformatically predicted gene-microRNA regulatory networks in high FLAIR signal GBMs.

microRNAs	genes	fold change	regulation
miR-219	**POSTN**	**7.45787885**	**up**
miR-127*	**CXCL12** *	**1.95140035**	**up**
miR-127	**COL1A1**	**1.69140875**	**up**
miR-127	**COL6A3**	**2.1281926**	**up**
miR-127	**GRB10**	**1.63224725**	**up**
miR-127	**SRPX2**	**1.78434725**	**up**
**miR-224**	AQP4	−1.841773	down
**miR-199b**	KIF1A	−1.8371131	down
**miR-223**	MPPED2	−2.12913255	down
**miR-223**	CTNNA2	−1.64381515	down
**miR-223**	PKP4	−1.59000765	down
**miR-223**	KIF5C	−1.54863265	down

*validated binding; bold text = upregulated.

Among the five concordant microRNA hits across the discovery and validation sets (Table S2), a gene target search demonstrated multiple bioinformatically predicted and inversely expressed genes in the high FLAIR patient group. There were two top downregulated microRNAs (Table S2). Interestingly, miR-219 was significantly downregulated in the Mesenchymal GBM subtype (P<0.0001, Figure 8d) and showed an inverse correlation with *POSTN* (Rsq = 0.205, Figure 8d) suggesting a potential miR-219-POSTN regulatory mechanism in GBM cellular invasion. Interestingly, the other top downregulated microRNAs in the high FLAIR group, miR-127, was experimentally shown to target CXCL12 mRNA [37], a major factor for GBM invasion [25], [38], [39], [40] and also one of our top genes accounting for high FLAIR volumes (Table 4 and S1).

## Discussion

In this study, we present the first comprehensive radiogenomic analysis using quantitative MRI volumetrics and large-scale gene- and microRNA expression profiling in GBM. We identified MRI-FLAIR as an imaging surrogate for GBMs highly enriched in genes and microRNAs involved in cellular migration/invasion and specifically identified miR-219 as a potential regulator of cellular invasion by binding to the 3′UTR of the *POSTN* gene, thus diminishing POSTN protein levels.

There was a slight positive correlation (Rsq = 0.13) (Figure 4a) between the volumes of contrast enhancement (active tumor) and peritumoral FLAIR signal abnormality (peritumoral edema and tumor infiltration). There was no correlation seen between the FLAIR and necrosis volumes (Figure 4b). These findings suggest that FLAIR, as an imaging surrogate for edema/invasion, captures a distinct aspect of tumor biology largely independent of tumor size and volumes of necrosis. Since areas of peritumoral FLAIR hyperintensity contain a mixture of both edema and cell invasion in GBM [19], [27], [38], [41], [42], [43], we can further hypothesize that high FLAIR radiophenotypes likely reflect a unique molecular tumor composition driving cellular migration and invasion.

Strengthening our above findings and hypothesis, we found in the high FLAIR patients that the top concordant genes and microRNAs across both the discovery and validation sets were highly associated with cancer, cellular movement, cell morphology, and cell signaling. A total of 29 of our top 53 genes were reported to play a role in cancer by IPA, while 20 were reported to play a role in cellular migration and invasion and 16 were reported to be involved in both cancer and invasion. This shows that by means of patient substratification into high and low FLAIR radiophenotypes, we are able to identify GBM subtypes with genes and microRNAs accounting for and possibly promoting migration and invasion. This was true for both mRNA and microRNA (Figure 5, 6, and 7; Table S1 and S2); across the discovery and validation sets, there were 5 concordant microRNAs. Despite this relatively low number, “cancer” and “cellular movement” were represented among the top diseases and cellular functions in IPA.

Interestingly, the top upregulated gene in both the discovery and validation sets was *POSTN* (Table S1), a gene previously implicated downstream of TWIST1 as being involved in invasion and mesenchymal transition in vitro [44]. Another report showed *POSTN* to be ranked among the top upregulated genes in ovarian cancer and GBM in both Agilent and Affymetrix microarray platforms [45].

In our study, *POSTN* expression was significantly associated with survival and time to disease progression in the TCGA database (P<0.0009; Figure 8a and 8b). When examining *POSTN* in the previously described GBM molecular subtypes [36], we found significant *POSTN* upregulation in the Mesenchymal compared to Proneural GBM subtype (P<0.0001; Figure 8c), confirming POSTN's possible role in mesenchymal transition. POSTN inducing cellular invasion through epithelial-mesenchymal transformation has been described in 293T kidney cell lines [46], [47]. Despite Mesenchymal and Proneural being some indicator for survival in GBM (P = 0.202; data not shown), *POSTN* was a stronger predictor and prognostic marker in the Cox proportional hazards model (*POSTN*: P = 0.03, GBM Subtype: P = 0.1; data not shown). This demonstrates that POSTN also captures a GBM subtype independent aspect of GBM invasion. Therefore, POSTN inhibition can be proposed to target mesenchymal transition and cancer cell invasion, two major causes of GBM aggressiveness and failure of current therapy.

The top downregulated microRNA across the discovery and validation sets was miR-219 (Table S2). Our data show that *POSTN* and miR-219 are inversely correlated (Rsq = 0.204) and that this is also true across the Mesenchymal and Proneural subtypes (P<0.0001; Figure 8d). Interestingly, miR-219 is known to have a potential binding site in the 3′UTR of the *POSTN* gene [35]. This suggests a potential role of miR-219 in downregulating *POSTN* in GBM mesenchymal transition and cellular invasion. Most importantly, this signature can be noninvasively detected by MRI upon initial patient consultation.

CXCL12 and CXCL14 were other top genes associated with the invasive radiophenotype in the high FLAIR volume group. CXCL12 is a known chemokine described in invasion and cell migration and is a ligand of CXCR4, a chemokine receptor that promotes tumor migration and invasion [25], [38], [39], [40]. McMillan et al. [25], [38] demonstrated an increased T2-signal intensity in GBM patients with high CXCR4 expression, further corroborating the validity of invasion associated genes in our study. Interestingly, miR-127, the second top downregulated microRNA in the high FLAIR group, was experimentally shown to target CXCL12 [35], [37]. This and the fact that other inversely correlated mRNAs and microRNAs with predicted binding sites (Table 4) were uncovered in our radiogenomics screen, further suggests a role of MRI as an early screening method for GBM specific molecular compositions and regulatory microRNA-mRNA mechanisms.

A limitation of the radiological data present in the TCGA is the lack of image-sample registration; thus, gene expression profiles cannot be matched to a specific location on MRI. Despite TCGA samples originating from the contrast enhancing core parts of the tumor, we showed a strong overrepresentation of migratory and invasive cell functions with higher peritumoral FLAIR volumes. Despite previous studies showing a different gene expression profile of core versus rim tumor parts [48], [49], [50], our analysis suggests that the potential of cellular migration and invasion may already be inherent in the GBM core and can be linked to the extent of peritumoral FLAIR signal abnormality. These findings are currently undergoing molecular mechanistic and imaging validation studies at our institutions. Certain limitations also exist with the use of microarray technology and analysis, e.g. due to the large number of probes, false positive gene hits may occur. However, using stringent false discovery rate correction, both validation and discovery sets, as well as careful literature cross-referencing, and microRNA target exploration increases robustness of findings. Finally, prospective validation is needed to fully establish MRI as a clinically applicable radiogenomics biomarker.

### Conclusion

MRI-FLAIR readily and noninvasively detected key cancer genomic components responsible for cellular migration and invasion in GBM. It further detected genes and microRNAs highly associated with mesenchymal transformation and invasion. As a discovery tool, MRI specifically identified *POSTN* and miR-219, suggesting a regulatory network promoting mesenchymal transition and invasion in GBM.

Since cellular invasion is a major cause of current treatment failure, surgical extent of resection and adjuvant treatment planning are of utmost importance. Here, we propose a novel diagnostic method to screen for cancer subtypes and molecular correlates of cellular invasion. Our findings also have potential therapeutic significance since successful molecular inhibition of invasion will improve therapy and patient survival in GBM.

## Supporting Information

Table S1 High FLAIR top concordant mRNAs in discovery and validation sets.
(TIF)

Table S2 High FLAIR top concordant microRNAs in discovery and validation sets.
(TIF)
