# Correction: Radiogenomic Mapping of Edema/Cellular Invasion MRI-Phenotypes in Glioblastoma Multiforme

**DOI:** 10.1371/annotation/b5267cb3-6aa7-47fc-a648-47f30a7cff3e

**Published:** 2012-02-01

**Authors:** Pascal O. Zinn, Bhanu Mahajan, Pratheesh Sathyan, Sanjay K. Singh, Sadhan Majumder, Ferenc A. Jolesz, Rivka R. Colen

There was a spelling error in the second author's name. The correct spelling is Bhanu Mahajan. The correct citation is: Zinn PO, Mahajan B, Sathyan P, Singh SK, Majumder S, et al. (2011) Radiogenomic Mapping of Edema/Cellular Invasion MRI-Phenotypes in Glioblastoma Multiforme. PLoS ONE 6(10): e25451. doi:10.1371/journal.pone.0025451 

