# Supplementary figures and images for: Radiogenomic Mapping of Edema/Cellular Invasion MRI-Phenotypes in Glioblastoma Multiforme
Source: PLoS One. 2011 Oct 5;6(10):e25451. doi: 10.1371/journal.pone.0025451 (PMC3187774; doi:10.1371/journal.pone.0025451)

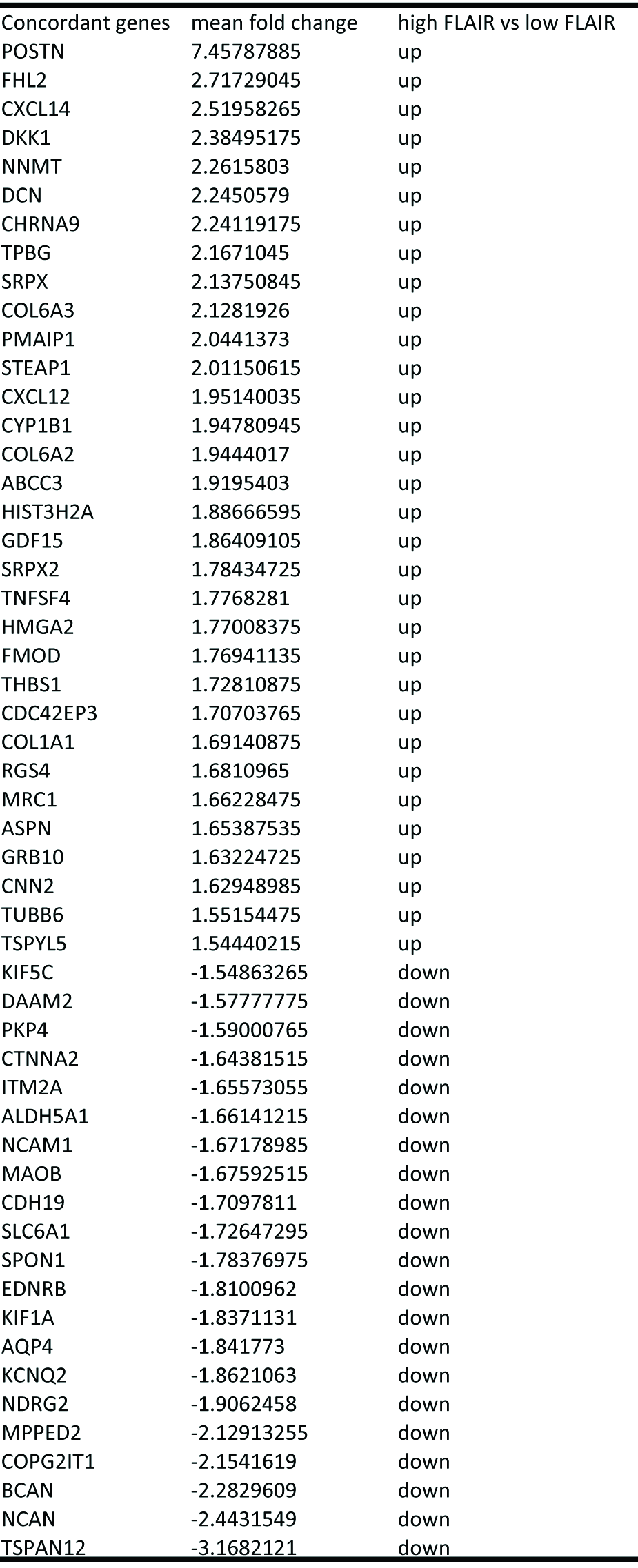

Supplement: Table S1 — High FLAIR top concordant mRNAs in discovery and validation sets. (TIF) [file pone.0025451.s001.tif]

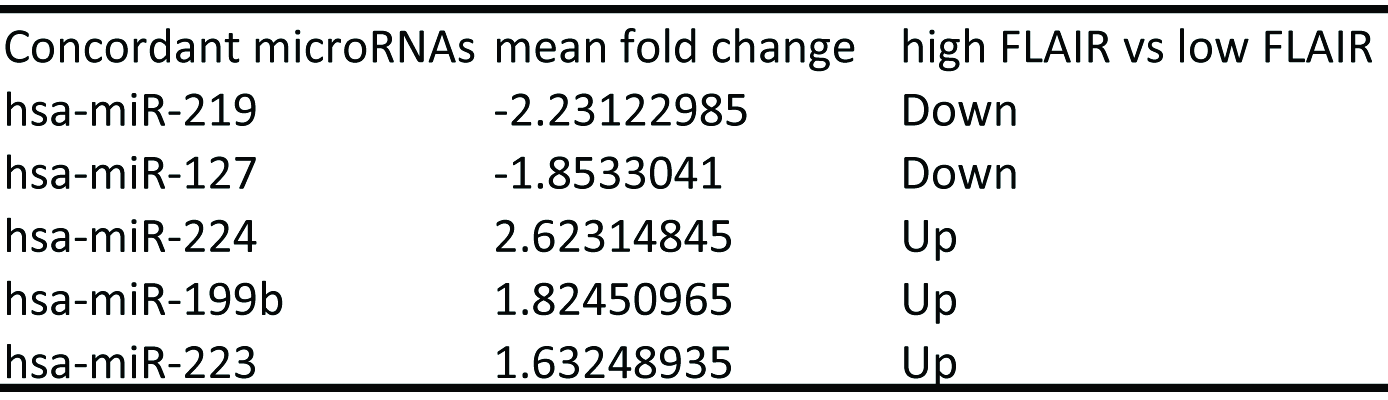

Supplement: Table S2 — High FLAIR top concordant microRNAs in discovery and validation sets. (TIF) [file pone.0025451.s002.tif]
